# The complete mitochondrial genome of *Sarotherodon galilaeus* (Linnaeus, 1758) (Perciformes: Cichlidae) and its phylogenetic placement

**DOI:** 10.1080/23802359.2021.1888327

**Published:** 2021-03-16

**Authors:** Congqiang Luo, Pinhong Yang, Suqin Wang

**Affiliations:** a Hunan Engineering Research Center of Aquatic Organism Resources and Environmental Ecology, Hunan University of Arts and Science, Changde, China; b Hunan Provincial Key Laboratory for Health Aquaculture and Product Processing in Dongting Lake Area, Hunan University of Arts and Science, Changde, China; c Hunan Provincial Collaborative Innovation Center for Efficient and Health Production of Fisheries, Hunan University of Arts and Science, Changde; d Changde Research Center for Agricultural Biomacromolecule, Hunan University of Arts and Science, Changde, China

**Keywords:** *Sarotherodon galilaeus*, mitochondrial genome, phylogenetic analyses

## Abstract

*Sarotherodon galilaeus* (Linnaeus, 1758), a cichlid species that is naturally distributed in African and Eurasian waters, was introduced in many Asian countries for aquaculture. To date, rare genetic studies focused on this species have hindered our understanding of this species. Here, we reported the complete mitochondrial genome of *S. galilaeus* that was sequenced using next-generation sequencing technology. The resulting mitogenome of *S. galilaeus* was 16,630 in length and comprised 13 protein-coding genes (PCG), 22 transfer RNA (tRNA) genes, 2 ribosomal RNA genes (rRNA), and one control region (D-loop). Phylogenetic analysis indicated that Oreochromini species contained two lineages (I and II) and *S. galilaeus* clustered with *Oreochromis aureus* rather than other *Sarotherodon* species.

*Sarotherodon galilaeus* (Linnaeus, 1758), a cichlid species that is naturally distributed in Africa and Eurasia, was introduced in many Asian countries for aquaculture, including in China (www.fishbase.org). Given that this species can tolerate hypersaline conditions, and can reproduce even in the high level of salinity, *S. galilaeus* was used to hybridize with other Oreochromini species (i.e. *Oreochromis niloticus*) for improving adaptive capacity (Yan and Wang 2010). To date, studies largely focused on the physiology and biology of *S. galilaeus* (Zeilstra et al. 2003; Yan and Wang 2010), whereas sparse genetic analyses have been conducted for this species. Therefore, this study reported the whole mitochondrial genome of *S. galilaeus* for the first time using next-generation sequencing technology and attempted to infer its phylogenetic status.

We collected the sample of *S. galilaeus* in July 2019 from Hengxian county (22.677 N, 109.266E), Guangxi Province, China. The sample (Voucher number: JLVLF2019001) was stored in the fish collection of Hunan University of Arts and Science. Total genomic DNA was extracted from fin tissues using a Genomic DNA Isolation Kit (QiaGene, Germany). We used the Illumina MiSeq platform to sequence the complete mitochondrial genome (Illumina Inc, San Diego, CA, USA) and assembled the raw sequence reads into contigs using SPAdes 3.9.0 (Bankevich et al. 2012). We finally obtained the complete mitochondrial genome using the contigs in SOAPdenovo (Luo et al. 2012) and annotated the protein-coding genes and rRNA genes using MITOS (Bernt et al. 2013).

The size of the *S. galilaeus* mitogenome was found to be 16,630 base pairs (bp) in length and comprised (*ND1*, *ND2*, *ND3*, *ND4*, *ND4L*, *ND5*, *ND6*, *COI*, *COII*, *COIII*, *ATP6*, *ATP8*, *Cyt b*), 2 rRNA genes (12S rRNA and 16S rRNA), 22 tRNA genes and a control region (D-loop) (GenBank nos: MW046257). The structural organization and gene order were in line with those in other typical teleosts. A total of 18 mitogenomes including 17 Oreochromini mitogenomes and one outgroup were aligned using MUSCLE (Edgar 2004). All 13 protein-coding genes were selected manually and then were combined into a single sequence for phylogenetic analyses. Maximum likelihood (ML) technique with nucleotide substitution model (GTR + I + G) was employed to construct trees. The ML tree was constructed in RAxML-VI-HPC (Stamatakis 2006) with 1000 nonparametric bootstrap replicates.

ML tree obtained two well supported major lineages with high supported values (Lineage I and II; Figure 1). Both clades I and II contained species from genus *Oreochromis*, implying taxonomic problems occurred in this genus. In addition, *S. galilaeus* was found to be grouped with *Oreochromis aureus*, rather than other *Sarotherodon* species (Figure 1). The result suggested genus *Sarotherodon* was not monophyletic taxa. Thus, more work on morphological features and phylogenetic inferences should stand together to resolve the complex relationships among Oreochromini species.

**Figure 1.**
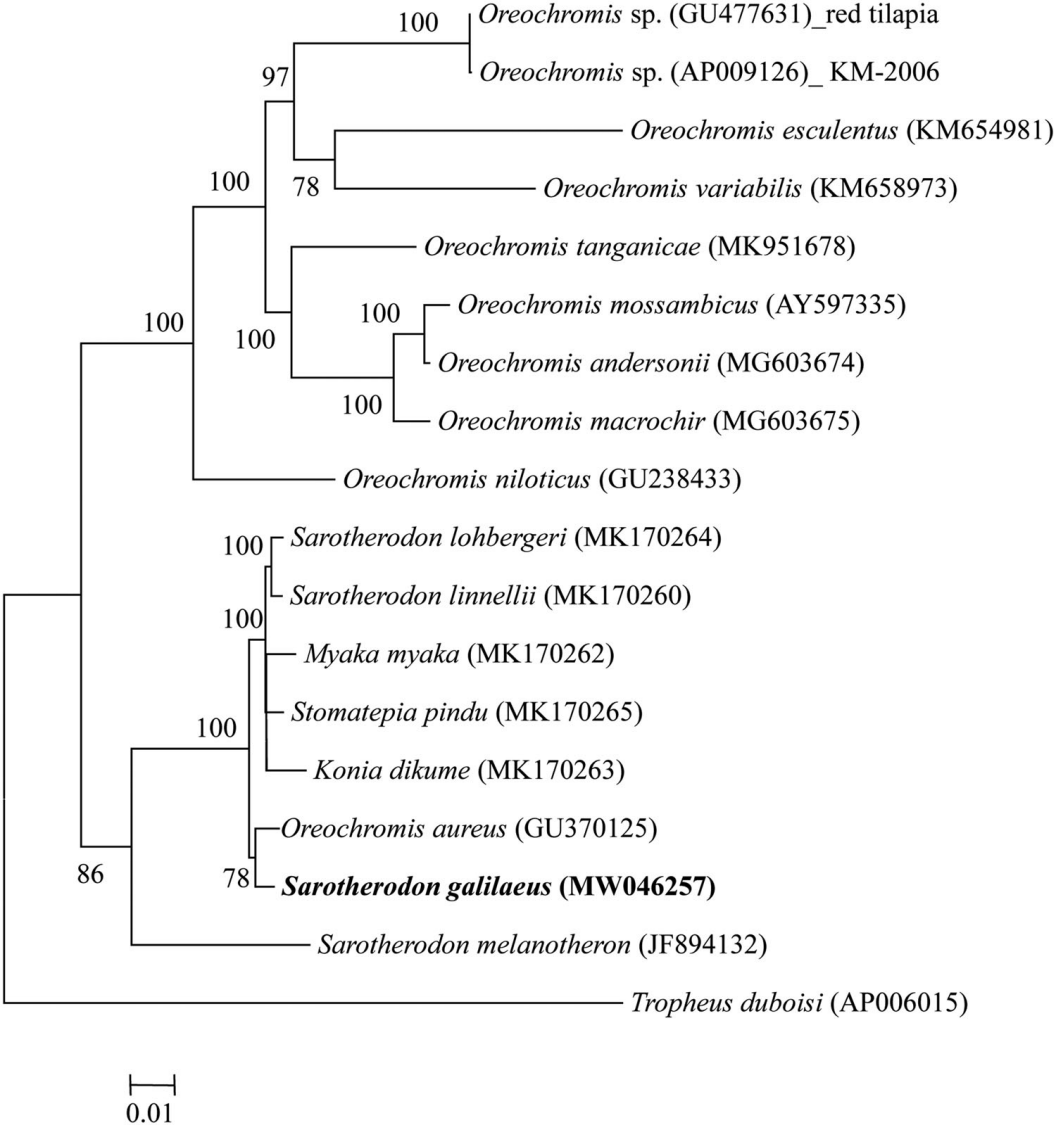
Phylogenetic trees based on maximum likelihood technique showing the phylogenetic relationships among 17 Oreochromini mitogenomes based on 13 protein-coding genes. Values on branches indicate bootstrap values from maximum likelihood analyses.

## Ethical approval

Experiments were performed in accordance with the recommendations of the Ethics Committee of Hunan University of Arts and Science. These policies were enacted according to the Chinese Association for the Laboratory Animal Sciences and the Institutional Animal Care and Use Committee (IACUC) protocols.

## Data Availability

The genome sequence data that support the findings of this study are openly available in GenBank of NCBI at (https://www.ncbi.nlm.nih.gov/) under the accession no. MW046257. The associated BioProject, SRA, and Bio-Sample numbers are PRJNA695435, SRR13570802 and SAMN17614357, respectively.
